# Dynamic changes in serum cytokine levels and their clinical significance in predicting acute GVHD

**DOI:** 10.18632/oncotarget.15738

**Published:** 2017-02-25

**Authors:** Chunyan Zhang, Wenrong Huang, Pengjun Zhang, Qingyi Zhang, Guanghong Guo, Feng Gu, Hua Yang, Yurong Wang, Xueliang Huang, Qian Jia, Yaping Tian

**Affiliations:** ^1^ Core Laboratory of Translational Medicine, State Key Laboratory of Kidney Disease, Chinese People's Liberation Army General Hospital, Beijing, China; ^2^ Department of Hematology, Chinese People's Liberation Army General Hospital, Beijing, China; ^3^ Department of Hematology, Air Force General Hospital, People's Liberation Army, Beijing, China; ^4^ Department of Clinical Biochemistry, Chinese People's Liberation Army General Hospital, Beijing, China

**Keywords:** allogeneic hematopoietic stem cell transplantation, graft-versus-host disease, cytokines, biochemical indices, aGVHD prediction

## Abstract

To explore the clinical significance of cytokines and biochemical tests in acute graft-versus-host disease (aGVHD), we detected the concentrations of 8 cytokines and 19 conventional biochemical markers in the sera of aGVHD and non-GVHD patients throughout the process of allogeneic hematopoietic stem cell transplantation and the onset of aGVHD. Predictive models were then established using the 27 indices, and models were verified by a prospective trial. The 27 indices showed significant differences between aGVHD patients and non-GVHD control subjects (two-tailed *p*<0.05) prior to transplantation and before the onset of aGVHD. Our models, established by binary logistic regression on days +7 and +14, showed a significant absolute capacity of predicting grade 2∼4 aGVHD with positive and negative predictive values of at least 70%. Our data showed that the progression of aGVHD could induce dynamic changes in the levels of serum cytokines and biochemical markers. Because most of these tests were less specific for aGVHD, these changes were easily neglected in clinical work. However, by combining cytokine and biochemical tests, the established prediction model can greatly improve the ability of these biomarkers to predict the development of aGVHD one or two weeks earlier.

## INTRODUCTION

The occurrence of aGVHD after transplantation is the greatest obstacle to the clinical application of allo-HSCT. The incidence of aGVHD reaches 40%∼60%, and the fatality rate of aGVHD is as high as 75%. Although the diagnosis of aGVHD onset depends mainly on clinical manifestations and tissue biopsy, research has shown that molecular and biological markers and noninvasive techniques have some usefulness for GVHD diagnosis [1]. Ferrara et al.[2] created an algorithm that included TNFR1, ST2, and Reg3α in a prognostic score for GVHD. We and others have reported clinical findings for the prediction and diagnosis of GVHD, such as the use of mass spectrometric (MS) profiling of urine[3], serum samples [4, 5], microRNA of peripheral blood [6, 7] and T cells [8].

Acute GVHD occurs most frequently within 100 days after transplantation, which traditionally has defined the acute form of the disease. Research has shown that lymphocytes from the graft, mainly T cells, identify host antigens [3]. The sensitization reaction leads to an attack on the target host organization directly or indirectly, and during the development of this disease, many cytokines are released into the bloodstream. Acute GVHD predominantly damages the skin, upper and lower gastrointestinal (GI) tract, and liver. Up to 30% of recipients of HSCT will develop aGVHD > grade 2 despite immunosuppressive prophylaxis [9, 10]. Thus, our goal is to explore the clinical significance of cytokines and biochemical indices in noncutaneous grade 2∼4 aGVHD and predict a model for aGVHD > grade 2.

The occurrence of a “cytokine storm” and oxidative stress in the process of aGVHD would cause unique patterns or profiles of protein expression related specifically to aGVHD. Meanwhile, high concentrations of cytokines and other associated proteins would accumulate not only in target organization but also in the blood, serum or plasma. Accordingly, many research studies have shown that TNF-α and IL-2 are important inflammatory cytokines involved in aGVHD [11, 12]. Several studies have demonstrated that some biochemical indices, such as cholinesterase (CHE), total protein (TP), and blood urea nitrogen (BUN), could contribute to the diagnosis of aGVHD [13, 14].

In this study, we detected the dynamic changes in the levels of interleukin-8 (IL-8), IL-10, soluble CD40 ligand (sCD40L), tumor necrosis factor-α (TNF-α), monocyte chemoattractant protein-1 (MCP-1), MIP-1α (CCL3), RANTES (CCL5), LIGHT (TNFSF14) and 19 other biochemical indices in serum from aGVHD patients and non-GVHD control subjects during the process of HSCT. We aimed to establish a noninvasive diagnostic method and a model to predict aGVHD using serum cytokines and biochemical markers.

## RESULTS

### Dynamic changes in the levels of cytokines and biochemical markers in serum from aGVHD patients and non-GVHD control subjects during HSCT

**Figure 1:**
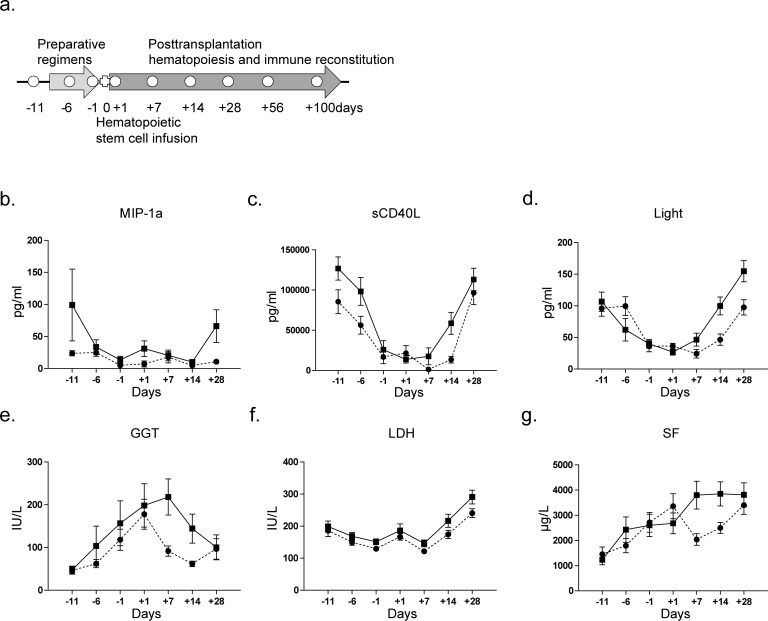
Blood specimen collection and dynamic changes in indices at the sequential time points during HSCT **a**. For non-aGVHD patients, peripheral blood specimens were collected on days −11, −1, +1, +7, +14, +28, +56, and +100; for aGVHD patients, two more blood specimens were collected, one at the onset of aGVHD and another when aGVHD was cured. **b**.∼**g**. Dynamic changes in MIP-1α, sCD40L, LIGHT, GGT, LDH and SF levels in serum from aGVHD and non-GVHD patients. The indices were compared by the Mann-Whitney U test. (●, non-GVHD; ■, aGVHD).

Most of our transplantation procedures comprised three periods: the preparative regimen (day −10∼day −3), hematopoietic stem cell infusion (day 0), and post-transplantation hematopoiesis and immune reconstitution (Figure 1a). The 20 aGVHD cases (grade 2∼4) in the training cohort showed a median time point of aGVHD occurrence of day +28 (range +15∼+70). Eight cytokines (IL-8, IL-10, sCD40L, TNF-α, MCP-1, MIP-1α, RANTES and LIGHT) and 19 biochemical indices (ALT, AST, GGT, ALB, TP, TB, DB, TBA, UN, Cr, UA, ADA, ApoAI, ApoC, SOD, SF, CHE, ALP and LDH) were analyzed in aGVHD patients and non-GVHD control subjects at each time point. As shown in Figure 1, Supplementary Figure 1 and Supplementary Figure 2, before pretreatment (on day −11), the levels of IL-10, TP and LIGHT in the sera of aGVHD patients were significantly different from those in the sera of the non-GVHD group (*p* < 0.05). More interestingly, we found that sCD40L, LIGHT, ALT, AST, GGT, TB, DB, TBA, ApoC, SOD and SF levels began to increase 1∼2 weeks prior to the clinical onset of aGVHD (on day +7 and +14). Because half of our aGVHD cases occurred within 28 days following HSCT, on day +28, the levels of many parameters changed significantly, including those of ALT, ADA, ALP, MIP-1α, LIGHT, and TNF-α. The differences between all indices were calculated by the Mann-Whitney U test (Supplementary Table 1). The dynamic levels of the 27 analyzed indices suggested that the status of the immune system and microenvironment in aGVHD patients is significantly different from that in non-GVHD subjects before pretreatment and prior to the diagnosis of aGVHD, indicating that the development of aGVHD can be predicted using serum indices only.

### Diagnostic values of different indices for predicting aGVHD

**Figure 2:**
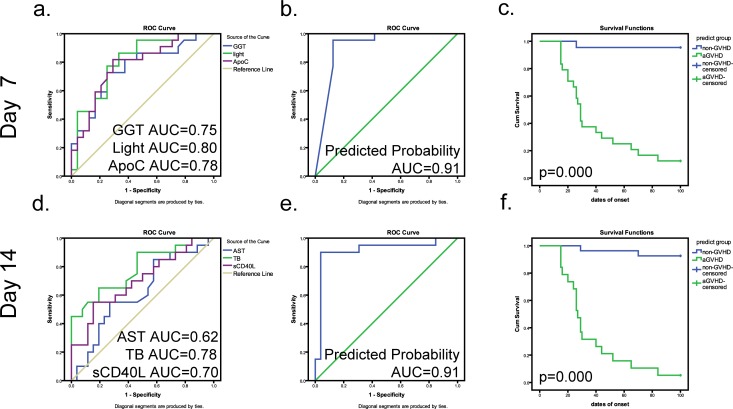
Predictive models on days +7 and 14 combining cytokines and biochemical criteria for grade 2∼4 aGVHD **a**., **d**. Respective ROC curves of the predictive values of differential indices on days +7 and +14 (GGT, blue line, AUC = 0.75; Light, green line, AUC = 0.80; ApoC, purple line, AUC = 0.78) (AST, blue line, AUC = 0.62; TB, green line, AUC = 0.78; sCD40L, purple line, AUC = 0.70). **b**., **e**. ROC curve of the cumulative probability of predictive model for aGVHD on days +7 and +14. **c**., **f**. The prediction rates for aGVHD of the combined model on days +7 and +14. In the Kaplan-Meier curves, blue represents a case predicted to be negative; green represents a case predicted to be positive. Tick marks indicate patients whose data were censored from further analysis at the initiation of aGVHD. Tick marks at 100 days indicate patients who were alive without aGVHD.

We further analyzed correlations of the 27 indices and established prediction models for aGVHD using binary logistic regression analyses on days −11, −1, +1, +7 and +14. The indices with a significant contribution to the model (*p* < 0.05) that were relatively independent of each other (r < 0.7, *p* < 0.01) were substituted into the binary logistic regression (method “Forward condition”). The models on days −11, −1, +7 and +14, especially days +7 and +14, showed a significant absolute capacity of predicting aGVHD (Figure 2 and Supplementary Figure 3). As shown in Figure 2a and 2b, three indices, GGT, LIGHT and ApoC, entered into the model at day +7, improving the AUC of binary logistic regression (AUC: 0.910, 95% CI: 0.817∼1.000). The cutoff value, sensitivity and specificity were 0.500, 95.45% and 87.50%, respectively. Moreover, the PPV and NPV values of the model reached 90.00% (18/20) and 96.00% (24/25), respectively (Figure 2c). Similarly, the model including AST, TB and sCD40L also showed a feasible effect for the prediction of aGVHD on day +14 (cutoff value: 0.508, sensitivity: 90.00%, specificity: 96.15%) (Figure 2e), with PPV and NPV values of 95.00% (19/20) and 92.00% (23/25), respectively (Figure 2f). The Kaplan-Meier curve showed that the models on day +7 and +14 could predict aGVHD occurrence after transplantation.

The classification equations combined with cytokines and biochemical indices for predicting grade 2∼4 aGVHD are as follows (P, predict possibility):

Day +7: Y = Logit (P/ (1-P)) = −426.635+1.692×GGT+7.427×ApoC+7.837×LIGHT

Day +14: Y = Logit (P/ (1-P)) = −10.158+0.152×AST+0.475×TB+0.001×sCD40L

### Diagnostic values of potential biomarkers in the development of aGVHD

**Figure 3:**
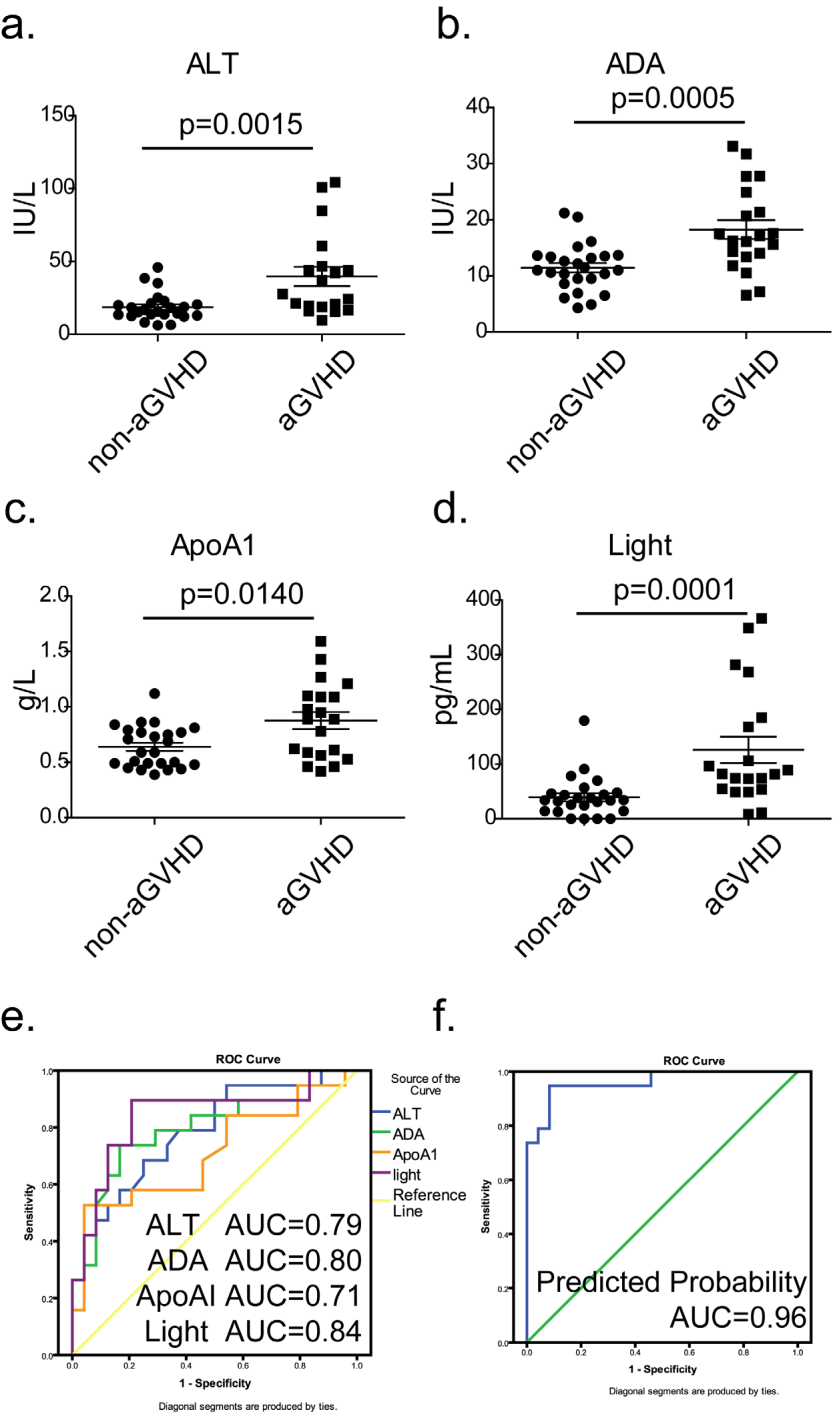
Diagnostic model combining cytokines and biochemical criteria at the onset of aGVHD **a**.∼**d**. Comparison of the expressions of ALT, ADA, ApoAI and LIGHT between patients in the non-GVHD (day +28) and aGVHD groups at the onset of aGVHD (●, non-GVHD; ■, aGVHD); *P* values were calculated using the Mann-Whitney U test. **e**. Respective ROC curves of diagnostic values of ALT (blue line), ADA (green line), ApoAI (orange line) and LIGHT (purple line). **f**. ROC curve of the cumulative probability of the diagnostic model for grade 2∼4 aGVHD.

To explore potential biomarkers for diagnosing aGVHD, the concentrations of differential indices were compared between aGVHD patients (at the onset of aGVHD, without medication) and non-aGVHD controls (on day +28). Seven indices that were significantly elevated in patients with aGVHD (Figure 3 and Supplementary Figure 4) and 4 biomarkers, ALT, ADA, ApoAI and LIGHT, entered a diagnostic model by binary logistic regression analysis. The AUCs of ALT, ADA, ApoAI and LIGHT were 0.785 (95% CI: 0.647 ∼0.923), 0.797 (95% CI: 0.657 ∼0.937), 0.708 (95% CI: 0.544 ∼872) and 0.840 (95% CI: 0.709 ∼0.971), respectively (Figure 3e). The formula of the diagnostic model is: Y = Logit (P/(1-P)) = −11.655+0.0872×ALT+0.201×ADA+5.151×ApoAI+0.036×LIGHT (Figure 3f), with an AUC, sensitivity and specificity of 0.961 (95% CI: 0.905 ∼1.000), 94.73% and 91.67%, respectively. Our results demonstrated that biochemical indices such as ALT, ADA and ApoA are clearly associated with aGVHD. Surprisingly, IL-10, which is thought to inhibit the synthesis of certain cytokines, was shown to be elevated in aGVHD patients (Supplementary Figure 4).

Previous studies have demonstrated that TNF-α would decrease after clinical treatment [15]. We compared differences in the indices before and after treatment in aGVHD patients using a paired *t*-test. The results indicate that ADA and LIGHT decreased in patients taking medication, possibly showing a trend similar to that of the TNF-α levels (Supplementary Figure 5).

### Clinical prospective trial to evaluate the values of prediction models on days +7 and +14

**Figure 4:**
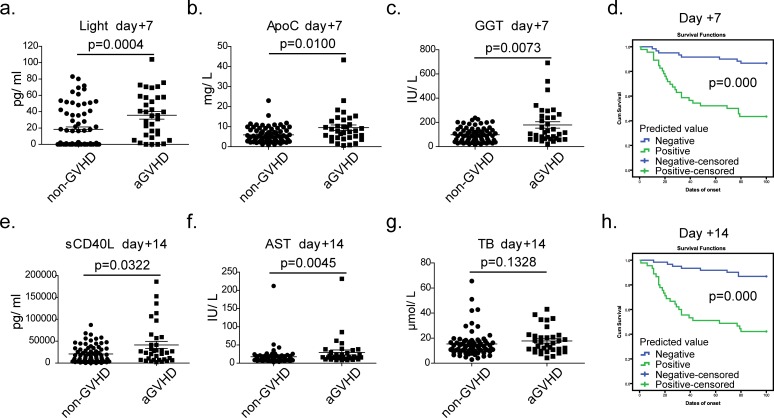
Verification of the predictive models using prospective experiments on days +7 and +14 **a**.∼**c**. The concentrations of GGT, ApoC and LIGHT in sera of validation cohort (●, non-GVHD, n = 72) ■, aGVHD, n = 34) at the day +7, *P* values were calculated using a Mann-Whitney test; **d**. The prediction rates for aGVHD of the combined model on days +7; **e**.∼**g**. The concentrations of AST, TB, and sCD40L in sera of validation cohort at the day +14; **h**. The prediction rates for aGVHD of the combined model on day +14. In the Kaplan-Meier curves, blue represents a case predicted to be negative; green represents a case predicted to be positive. Tick marks indicate patients whose data were censored from further analysis at the onset of aGVHD. Tick marks at 100 days indicate patients who were alive without aGVHD.

To assess the clinical value of our prediction models, 106 disease-free patients who had undergone allo-HSCT were monitored as a validation group to evaluate the predicted values of our models. On day +7 after transplantation, we detected the concentrations of GGT, ApoC and LIGHT in serum (Figure 4a∼4c) and then substituted the values into the formula. The resulting predictive possibility value, which was larger than the cutoff value (0.50, shown in Supplemental Table 2), was defined as positive and was associated with a high risk of mortality. Similarly, we evaluated the concentrations of AST, TB, and sCD40L on day +14 (Figure 4e∼4g) and substituted the values into the formula. The 106 patients were monitored for 100 days after transplantation. We next contrasted the predicted values with the actual incidence using Kaplan-Meier curves. As shown in Figure 4d, the PPV and NPV of the model on day +7 were 76.47% (26/34) and 72.22% (52/72), respectively, with an accuracy of 73.58%. On day +14 (Figure 4h), the PPV, NPV and accuracy of the model were 76.47% (26/34), 73.61% (53/72) and 74.53%, respectively. The total accuracy of the two models was 87.7% (93/106). Among the 13 missing cases, 10 cases were identified as false positive and exhibited severe infection; 3 cases exhibited severe aGVHD (3∼4 degrees) over a short time (+10∼+15 day), had a poor prognosis and were identified as false negative. These results demonstrated that our models were sensitive to the body's immune response and can be used as effective tools for predicting aGVHD.

## DISCUSSION

Although the diagnosis of aGVHD onset depends mainly on clinical manifestations and tissue biopsy, research has shown that molecular and biological markers and noninvasive techniques have some usefulness for GVHD diagnosis [2, 16]. Acute GVHD symptoms are the result of a complex set of interactions among cellular and soluble factors [17]. Meanwhile, the levels of cytokines and proteins produced by damaged recipient tissues increase in the circulatory system, creating a special plasma proteomic profile. The current study was designed to explore the clinical significance of cytokines and biochemical tests in patients with aGVHD.

We conducted a prospective study of plasma markers of aGVHD, especially cytokines and routine biochemical indices, to assess their predictive and diagnostic values for aGVHD. In the training group, our models show an excellent capacity of predicting grade 2∼4 aGVHD on day +7 (sensitivity: 95.45%, specificity: 87.50%, PPV: 90%, NPV: 96%) and +14 (sensitivity: 90.0%, specificity: 96.15%, PPV: 95%, NPV: 92%) following HSCT. In the prospective validation group, PPV and NPV in the model on day +7 are 76.47% and 72.22%, respectively, with an accuracy of 73.58%. On day +14, the PPV, NPV and accuracy of the model are 76.47% (26/34), 73.61% (53/72) and 74.53%, respectively. The decline in the overall sensitivity and specificity from the training set to the validation set findings may be caused by the randomness of the prospective trial. In conclusion, our prediction model was based on the view that cytokines and biochemical indices could predict aGVHD occurrence after transplantation.

We found that infection and inflammation were a significant source of false-positive reactions. Among the 10 false-positive cases, 3 patients developed bacteremia, 4 developed a cytomegalovirus infection, one developed gastrointestinal bleeding, one developed a pulmonary infection and one developed drug-induced liver damage. All three false-negative cases occurred during severe aGVHD (3∼4 degrees) over a short time period (+10∼+15 day), and all patients had a poor prognosis. The early administration of medication and low immunity might have caused the false negatives.

Hepatic complications are among the leading symptoms of aGVHD. In our study, biochemical indices that entered our prediction models, such as GGT, AST, TB and ApoC, reflect liver function and lipid metabolism in the body. It has been reported in previous studies that hyperbilirubinemia after allogeneic HSCT is associated with aGVHD processes, increased mortality and a worse prognosis [18]. The 2005 criteria of the National Institutes of Health (NIH) also show that TB (total bilirubin) > 2 times the upper limits of normal is one of the grading criteria of GVHD [19]. In the current study, we illustrated the dynamic changes in TB in both the aGVHD and non-GVHD groups (Supplementary Figure 2d); there were significant differences between the groups on day +7 and +14. Interestingly, we also illustrated the dynamic changes in DB (direct bilirubin) and TBA (total bile acid) levels for the first time and found the same tendency in the two indices. TB consists of DB and free bilirubin, supporting evidence for distinguishing different hepatic lesions. TBA is a sensitive indicator for liver cell necrosis and increases during even minor injuries [20]. At days +7 and +14, the elevations of TB, DB, TBA, and ALT (Supplementary Figure 2a) reflect more on liver damage in early post transplantation than liver aGVHD. The results indicated that hepatocyte injury would increase the risk of aGVHD through releasing cytokines, and more attention should be given to liver function in early post transplantation.

Research has shown that TNF-α is an important inflammatory cytokine involved in aGVHD. The level of TNF-α increases before aGVHD onset and decreases after treatment with medications such as steroids and pentoxifylline [15]. Therefore, we detected the level of TNF-α as a positive control. Interestingly, LIGHT and sCD40L showed better predictive ability for aGVHD on days +7 and +14, respectively. LIGHT, a newly identified member of the TNF superfamily (TNFSF14), is predominantly expressed by T cells [21]. LIGHT has been shown to stimulate the proliferation of T cells and trigger apoptosis in various tumor cells [22]. Geriel [23] proposed that LIGHT could inhibit the cytotoxic T lymphocyte response and was critical for optimal CD4+ T cell alloresponses in MHC class II-disparate GVHD in a mouse model. Our results confirmed that LIGHT was hypersensitive to the progression of aGVHD in humans and could predict aGVHD as a stable biomarker in serum. sCD40L is a platelet-derived mediator that may promote inflammation and endothelial cell dysfunction [24]. In a mouse model, Koji el. found that the infusion of a mAb against CD40L further increases the efficacy of lymphotox in β receptor-Ig, preventing GVHD [25]. These biomarkers are measurable in serum and showed an increasing trend before the clinical onset of aGVHD in our findings.

At the onset of aGVHD, patients have significantly higher levels of LIGHT and TNF-α. Paradoxically, IL-10, which is thought to inhibit the synthesis of inflammatory cytokines such as IL-8 and TNF-α, was elevated in patients with acute GVHD in our results. This finding indicates that IL-10 is produced in response to significant inflammation. Other inflammation-related cytokines, including IL-8, RANTES and MCP-1, showed no significant correlation with the development of aGVHD. After effective treatment, the levels of ADA, TNF-α and LIGHT decreased and showed an obvious correlation with the progression of aGVHD.

In conclusion, our results showed that the progression of aGVHD has some related variation in serum cytokines and biochemical tests. Furthermore, the established prediction model, by combining the status of certain cytokines and biochemical tests, warns of the earlier occurrence of aGVHD.

## MATERIALS AND METHODS

### Patients and serum samples

The serum samples were collected from 151 patients undergoing allogeneic stem cell transplantation at the Chinese PLA General Hospital between 2012 and 2015. A total of 45 cases (20 grade 2∼4 aGVHD and 25 non-aGVHD patients) were used for investigation and modeling, and 106 cases were used for validation. Acute GVHD was staged and graded using established criteria[26], and the clinical data are outlined in Table 1. Only serum samples that would have been discarded were used following informed consent from the patients. All procedures followed were in accordance with the ethical standards of the Ethics Committee of Chinese PLA General Hospital (Ref: 2013-32) and with the Helsinki Declaration of 1975, as revised in 2008. All experimental protocols were approved by the Ethics Committee of Chinese PLA General Hospital (Ref: 2013-32).

**Table 1:** Demographic and Clinical Characteristics of Patients with and without acute GVHD

	Discovery and Training cohort, N=45			Validation cohort, N=106			
	Non-GVHD, N=25	aGVHD, N=20	*P value	Non-GVHD, N=72	aGVHD, N=34	*P value	#*P* value
**Age, y**							
Median	31	39	0.167	38	35	0.408	0.692
Range	8-50	15-53		15-61	16-53		
**Gender**							
Male	14	22	0.157	51	21	0.379	0.158
Female	6	3		21	13		
**Diagnostic, % (no.)**							
AML	17.8(8)	20.0(9)	0.534	29.2(31)	9.4(10)	0.112	0.026
ALL	26.7(12)	11.1(5)		13.2(14)	14.2(15)		
CML	4.4(2)	6.7(3)		3.8(4)	1.9(2)		
MPD	6.7(3)	6.7(3)		12.3(13)	2.8(3)		
AA	0	0		2.8(3)	0.9(1)		
HL, NHL	0	0		6.6(7)	2.8(3)		
**Donor type, % (no.)**							
Matched related	15.6(7)	13.3(6)	0.300	30.2(32)	12.3(13)	0.106	0.210
Matched unrelated	15.6(7)	6.7(3)		5.7(6)	1.9(2)		
Mismatched related	24.4(11)	24.4(11)		32.1(34)	17.9(19)		
**Conditioning regimen intensity, %(no.)**							
Bu/Cy or Bu/Cy+ATG	22.2(10)	33.3(15)	0.701	46.2(49)	28.3(30)	0.061	0.351
Flu/Bu or Flu/Bu+ATG	17.8(8)	0		8.5(9)	0.9(1)		
TBI+Cy or TBI+Cy+ATG	15.6(7)	11.1(5)		13.2(14)	2.8(3)		
**Conditioning regimen intensity, based on ATG therapy, %(no.)**							
Bu/Cy or Flu/Bu or TBI+Cy	15.6(7)	13.0(6)	0.571	30.2(32)	12.3(13)	0.348	0.133
Bu/Cy+ATG or Flu/Bu+ATG or TBI+Cy+ATG	40(18)	31.1(14)		37.7(40)	19.8(21)		
**Grade at GVHD onset, % (no.)**							
0∼1	55.6(25)	0		67.9(72)	0		0.411
2	0	22.2(10)		0	20.8(22)		
3∼4	0	22.2(10)		0	13.2(14)		
**Organ target at GVHD onset, % (no.)**							
Skin	0	10.0(2)		0	5.9(2)		0.275
Gut	0	40.0(8)		0	35.3(12)		
Liver	0	30.0(6)		0	50.0(17)		
Combined	0	20.0(4)		0	8.8(3)		
**Attack time (day)**							
Median		28			33		0.947
Range	>100	15-70		>100	2-100		

Abbreviations: AML, acute myelocytic leukemia; ALL, acute lymphoblastic leukemia; CML, chronic myelogenous leukemia; MPD, myeloid proliferation disorders; AA, aplastic anemia; HL, Hodgkin's lymphoma; NHL, non-Hodgkin's lymphoma; Bu, busulfan; Cy, cyclophosphamide; Flu, fludarabine; ATG, antithymocyte globulin; TBI, total body irradiation.

*P values for difference between aGVHD and non-GVHD were calculated using a 2-sided χ^2^ test.

#*P* values for difference between training and validation sets were calculated using *t*-test or Mann-Whitney U test.

Most of our transplantation procedures consisted of three periods: the preparative regimen (day −10∼ day −3), hematopoietic stem cell infusion, and post-transplantation hematopoiesis and immune reconstitution (Figure 1a). Preparative regimens mainly included 6 schemes: (1) Bu/Cy scheme; (2) Bu/Cy+ATG (antithymocyte globulin) scheme; (3) Flu/Bu scheme; (4) Flu/Bu+ATG scheme; (5) TBI (the total body irradiation) +Cy scheme; and (6) TBI+Cy+ATG scheme. Based on the clinical diagnosis, matched type and patient's condition (age, physical condition), different preparative regimens would be given. In this study, most of the patients accepted the Bu/Cy scheme (Maryland 1 mg/(kg.6 h) × 3 days, cytarabine 2 g/m^2^, cyclophosphamide (Cy) 60 mg/(kg.d) × 2 days, methyl-ccnum-ccnu (Me-CCNU) 250 mg/m^2^)) or TBI (6∼10 GY/2d) plus Cy 60 mg/(kg.d) scheme. GVHD prophylaxis consisted of cyclosporine, methotrexate, and mycophenolatemofetil. The diagnosis of aGVHD was made based on the 2005 criteria of the National Institutes of Health (NIH) [13]. For non-aGVHD patients, peripheral blood specimens were collected on days −11, −1, +1, +7, +14, +28, +56, and +100. For aGVHD patients, two additional blood specimens were collected: one at the onset of aGVHD (patients were not taking medications), and another after aGVHD had resolved (usually 28 days later).

Serum samples were processed by centrifugation at 4000 rpm for 7 min at 25°C and were frozen in aliquots of 200μl at −80°C immediately for future use. No freeze-thawing was allowed prior to cytokine detection.

### Multiplex microbeadimmunoassay

The serum concentrations of IL-8, IL-10, and sCD40L, TNF-α, MCP-1, MIP-1α, and RANTES were detected using a human cytokine/chemokine magnetic bead immunoassay kit (HCYTOMAG-60K, Millipore, Billerica, MA) according to the instructions of the manufacturer. Briefly, 25μL of the serum sample (1:100 dilution for RANTES) and 25μL of premixed beads that have been covered by specific capture antibodies, were added to each well. After incubation with agitation overnight at 4°C, the plate was washed twice using washing buffer. Then, a 25μL cocktail of biotinylated detection antibodies was added to each well. After incubation with agitation at room temperature for 1 hour, 25μL of streptavidin-phycoerythrin was added. After another 30 minutes, the plate was washed twice with washing buffer. Finally, 150μL of sheath fluid were added to all wells, and the fluorescent signal of the beads was read using a Luminex^200^apparatus (Luminex, Austin, TX).

The concentration of LIGHT was detected using a human LIGHT/TNFSF14 Quantikine ELISA Kit (#DLIT00, R&D systems, Minneapolis, MN) according to the instructions of the manufacturer. A serum sample (50μL) was added to each well, and the values of absorbance were read using a microplate reader (Bio-Rad, Hercules, USA) set to 450nm.

We established each standard curve by 7 different concentrations and analyzed the median fluorescent intensity data for calculating cytokine concentrations in samples.

### Biochemical indices detection

The concentrations of alanine aminotransferase (ALT), aspartate aminotransferase (AST), gamma-glutamyltransferase (GGT), albumin (ALB), total protein (TP), total bilirubin (TB), direct bilirubin (DB), total bile acid (TBA), urea nitrogen (UN), creatinine (Cr), uric acid (UA), adenosine deaminase (ADA), apolipoprotein AI (ApoAI), apolipoprotein C (ApoC), superoxide dismutase (SOD), serum ferritin (SF), cholinesterase (CHE), alkaline phosphatase (ALP), and lactate dehydrogenase (LDH) were detected by a Roche Modular DDP automatic biochemical analyzer (Roche Diagnostics, Mannheim, Germany) and a Roche Modular E170 automatic immunity analyzer (Roche).

### Statistical analysis

The concentrations of the cytokines and biochemical indices in aGVHD patients and non-GVHD control subjects were compared by the Mann-Whitney U test. A 2-sided χ2 test was used to compare the differences in clinical patient characteristics. A binary logistic regression model was established to evaluate the binary diagnostic value and receiver operating characteristics (ROC) curves were established to evaluate the diagnostic value. The Kaplan-Meier curves were analyzed to estimate the prediction rate for aGVHD. The indices with a *p*-value of < 0.05 were confirmed to be significantly different. All statistical analyses were performed using SPSS software (ver.19.0), and graphs were generated using GraphPad Prism software (ver.6.0).

## SUPPLEMENTARY MATERIAL FIGURES AND TABLES


