# Supplementary material for: Dynamic changes in serum cytokine levels and their clinical significance in predicting acute GVHD
Source: Oncotarget. 2017 Feb 25;8(32):53691–700. doi: 10.18632/oncotarget.15738 (PMC5581142; doi:10.18632/oncotarget.15738)
Supplement: Supplementary file 1 [file oncotarget-08-53691-s001.pdf]

Dynamic changes in serum cytokine levels and their clinical significance in predicting acute GVHD

Supplementary Material

Supplementary Table 1. Serum indicators independent sample t test analysis

Day	Index*	Disease	N	Mean	Std.Error	Sig. (2-tailed)
-11	TP	Non-GVHD	20	64.855	1.119	0.038
		aGVHD	17	61.312	1.204	0.038
	IL-10	Non-GVHD	25	4.470	1.908	0.049
		aGVHD	20	0.129	0.071	0.032
	LIGHT	Non-GVHD	25	140.453	14.731	0.035
		aGVHD	20	94.864	14.384	0.032
-6	TBA	Non-GVHD	13	3.423	0.530	0.025
		aGVHD	15	6.747	1.216	0.021
	IL-8	Non-GVHD	23	67.664	9.160	0.016
		aGVHD	20	38.118	6.845	0.014
	LIGHT	Non-GVHD	21	138.875	23.160	0.045
		aGVHD	20	77.371	18.319	0.044
-1	SOD	Non-GVHD	25	170.620	8.883	0.059
		aGVHD	19	212.732	22.024	0.089
	LDH	Non-GVHD	25	129.664	6.605	0.057
		aGVHD	19	151.132	9.116	0.065
	LIGHT	Non-GVHD	24	60.496	12.858	0.040
		aGVHD	18	26.581	6.650	0.025
+1	CEH	Non-GVHD	25	5380.560	331.545	0.026
		aGVHD	20	4365.346	275.810	0.023
	MIP-1α	Non-GVHD	24	7.259	4.613	0.036
		aGVHD	20	30.096	10.009	0.047
	LIGHT	Non-GVHD	25	70.242	13.490	0.000
		aGVHD	20	6.915	2.051	0.000
+7	ALT	Non-GVHD	25	20.208	2.270	0.013
		aGVHD	20	41.927	8.734	0.024
	GGT	Non-GVHD	24	91.517	11.935	0.004
		aGVHD	20	218.095	42.214	0.008
	TB	Non-GVHD	25	16.096	1.229	0.001
		aGVHD	20	26.423	2.674	0.001
	DB	Non-GVHD	25	7.881	0.810	0.002
		aGVHD	20	14.755	2.067	0.004
	ApoC	Non-GVHD	25	6.226	0.665	0.003
		aGVHD	20	11.712	1.757	0.007

	SF	Non-GVHD	25	2036.538	229.041	0.003
		aGVHD	20	3795.955	551.954	0.006
	LDH	Non-GVHD	25	121.373	6.888	0.049
		aGVHD	20	146.264	10.647	0.057
	LIGHT	Non-GVHD	25	18.841	5.470	0.008
		aGVHD	22	45.762	8.428	0.011
+14	AST	Non-GVHD	25	18.435	3.485	0.045
		aGVHD	20	21.635	3.375	0.045
	GGT	Non-GVHD	25	61.773	6.350	0.010
		aGVHD	20	144.355	33.880	0.026
	TB	Non-GVHD	25	10.931	0.681	0.000
		aGVHD	20	19.970	2.462	0.002
	DB	Non-GVHD	25	5.481	0.536	0.004
		aGVHD	20	10.760	1.853	0.012
	SOD	Non-GVHD	25	152.915	10.361	0.014
		aGVHD	20	219.150	26.492	0.028
	SF	Non-GVHD	25	2499.038	216.667	0.008
		aGVHD	20	3846.300	478.626	0.016
	LDH	Non-GVHD	25	174.083	12.587	0.071
		aGVHD	20	216.560	20.497	0.087
	sCD40L	Non-GVHD	25	15971.256	5080.767	0.022
		aGVHD	20	45496.257	12553.136	0.039
	LIGHT	Non-GVHD	25	42.777	7.249	0.001
		aGVHD	19	101.711	17.451	0.005
+28	ApoC	Non-GVHD	25	8.528	0.737	0.017
		aGVHD	20	14.852	2.551	0.023
	LDH	Non-GVHD	25	240.428	13.603	0.050
		aGVHD	20	290.727	21.514	0.054
	IL-8	Non-GVHD	25	35.227	4.685	0.006
		aGVHD	20	86.527	17.689	0.008
	MIP-1α	Non-GVHD	25	11.507	3.432	0.007
		aGVHD	20	93.704	29.545	0.010
	TNFα	Non-GVHD	25	13.333	1.683	0.004
		aGVHD	20	27.603	4.556	0.006
	LIGHT	Non-GVHD	25	95.275	11.204	0.001
		aGVHD	20	166.596	16.397	0.001

*Comments: the units, LDH, ALT, AST, GGT, SOD, CEH (IU/L); ApoC (mg/L); TP (g/L); TBA, TB, DB (μmol/L); SF (μg/L); LIGHT, IL-8, IL-10, MIP-1α, sCD40L, TNFα (pg/mL)

Supplemental Table 2. Parameters in predict models for aGVHD and non-GVHD

Day	Indexs	ROC curve				Survival curve	
		Cutoff	AUC	Sensitivity (%)	Specificity (%)	Positive predictive value (%)	Negative predictive value (%)
-11	TP	62.15	0.699	85.00	58.82		
	LIGHT	115.26	0.735	75.00	88.24		
	Predicted probability	0.60	0.77	52.94	90.00	70	72
-1	SOD	193.20	0.617	47.37	80.00		
	LDH	127.10	0.655	84.21	60.00		
	Predicted probability	0.64	0.72	42.11	100.00	60	64
+1	IL-8	20.07	0.597	83.33	47.62		
	CHE	4077.10	0.687	83.33	61.90		
	Predicted probability	0.46	0.69	80.95	54.17	60	64
+7	GGT	115.25	0.755	72.73	75.00		
	LIGHT	18.04	0.805	86.36	66.67		
	ApoC	7.31	0.777	81.82	70.83		
	Predicted probability	0.50	0.91	95.45	87.50	90	96
+14	AST	16.80	0.616	55.00	73.08		
	TB	13.95	0.784	65.00	80.77		
	sCD40L	18378.00	0.700	55.00	84.62		
	Predicted probability	0.51	0.91	90.00	96.15	95	92

Figure Legends for Supplementary Figures

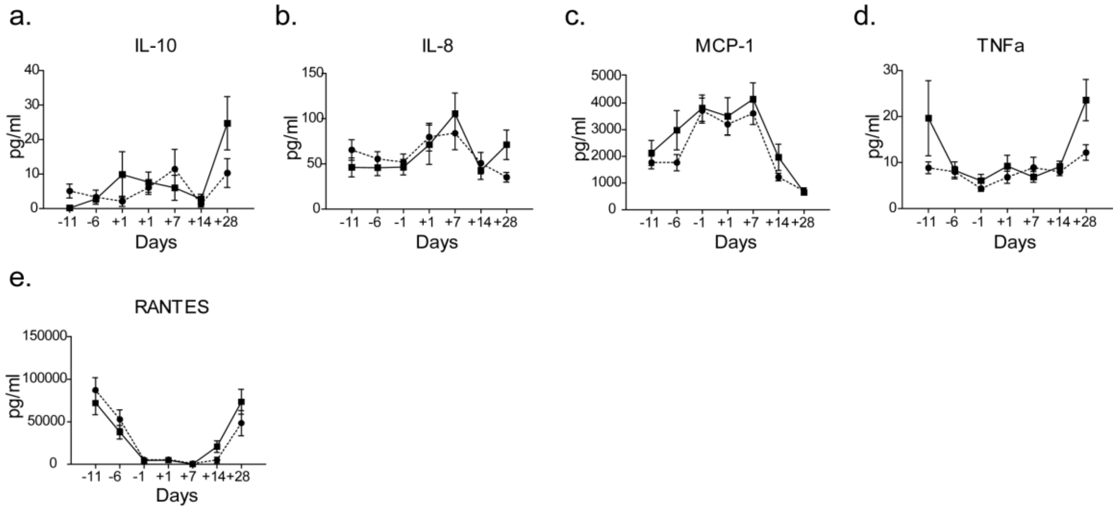

Supplementary Figure 1. Dynamic changes of cytokines in the sera from aGVHD patients and non-GVHD controls during HSCT. (●, non-GVHD; ■, aGVHD)

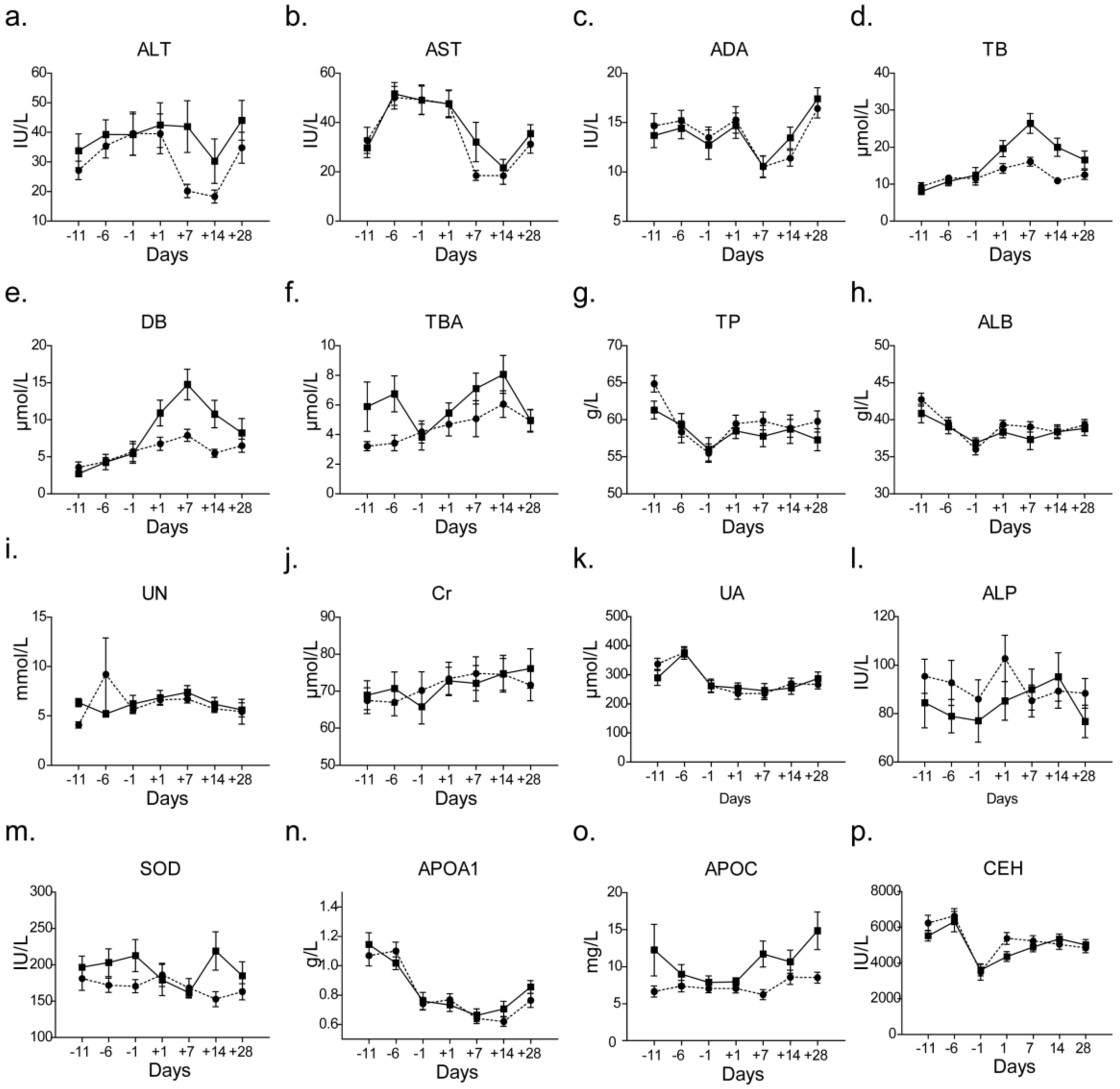

Supplementary Figure 2. Dynamic changes of biochemical criterions in the sera from aGVHD patients and non-GVHD controls during HSCT. (●, non-GVHD; ■, aGVHD)

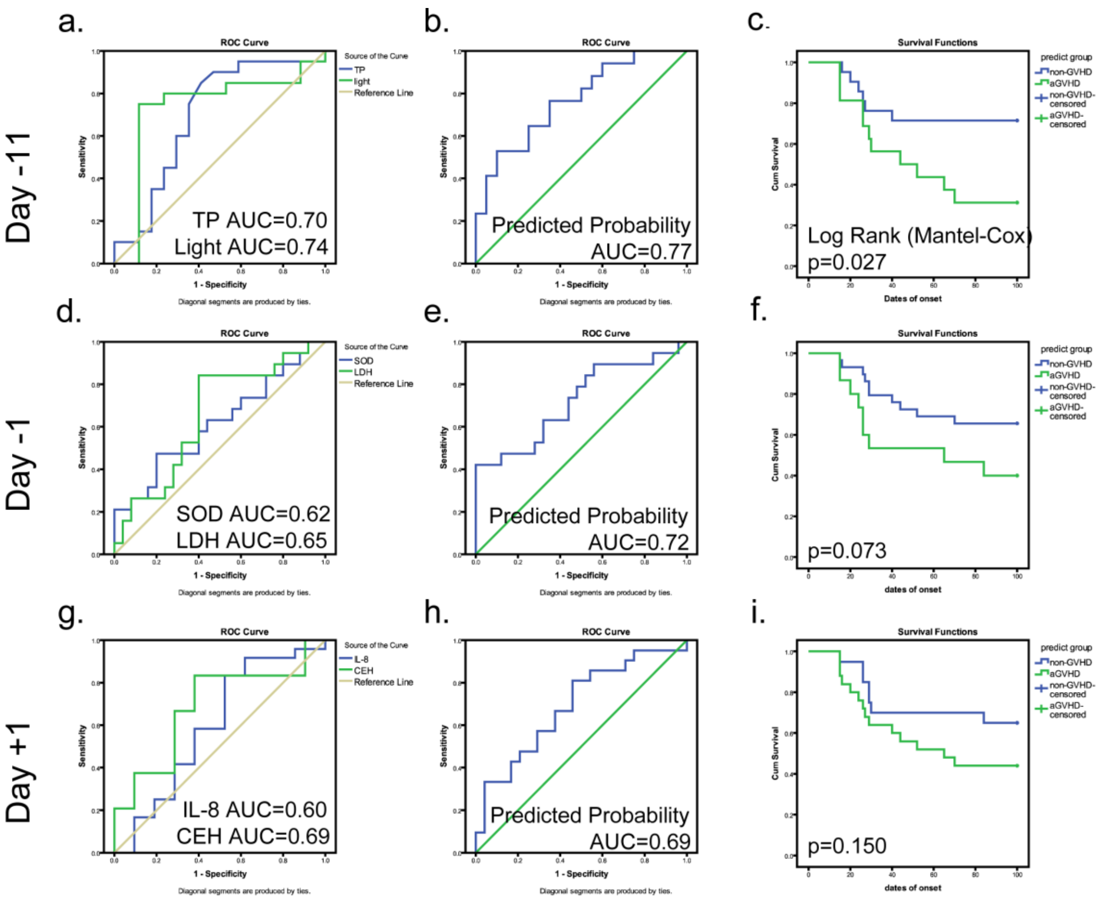

Supplementary Figure 3. Predictive models at day -11, -1 and +1 combining cytokines and biochemical criterions for grade 2~4 aGVHD. a, d, g. Respective ROC curves of predictive values of differential indicators at the day -11, -1, and +1; b, e, h. ROC curve of the cumulative probability of predictive model for aGVHD at the day -11, -1 and +1; c, f, i. The prediction rates for aGVHD of combined model at the day -11, -1 and +1. The classification equations combined with cytokines and biochemical indicators for predicting grade 2~4 aGVHD are as follows (P, predict possibility): Day -11: Y= Logit(P/ (1-P)) = 10.954-0.156×TP-0.010×LIGHT; Day -1: Y= Logit(P/ (1-P)) = -5.207+0.011×SOD+0.021×LDH; Day +1: Y= Logit(P/ (1-P)) = 4.992-0.001 ×CHE-0.012×IL-8. In the Kaplan–Meier curves, blue represents for case predicted to be negative, green represents for case predicted to be positive. Tick marks indicate patients whose data were censored from further analysis at aGVHD breakout. Tick marks at 100 days indicate patients who were alive without aGVHD.

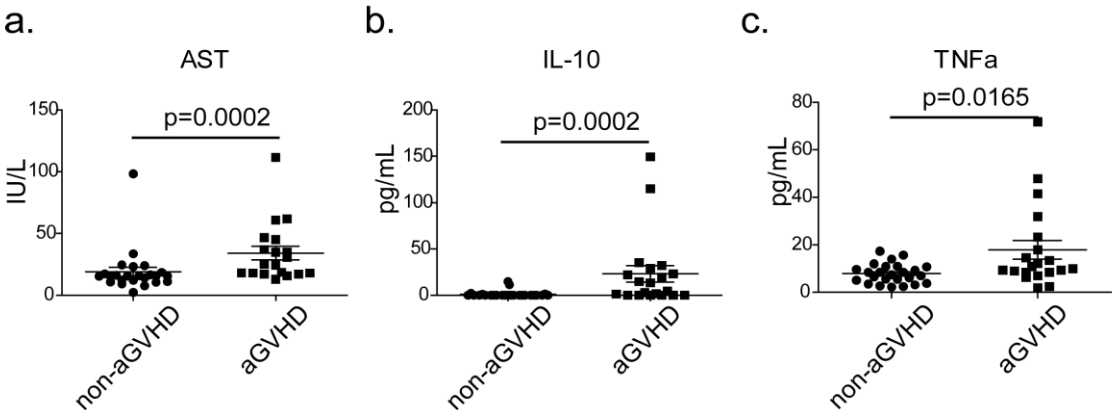

Supplementary Figure 4. The expressions of ALT(a), IL-10(b) and TNF-α(c) between aGVHD and non-GVHD group at onset of aGVHD. *P* values were calculated using a Mann-Whitney test. (●, non-GVHD; ■, aGVHD)

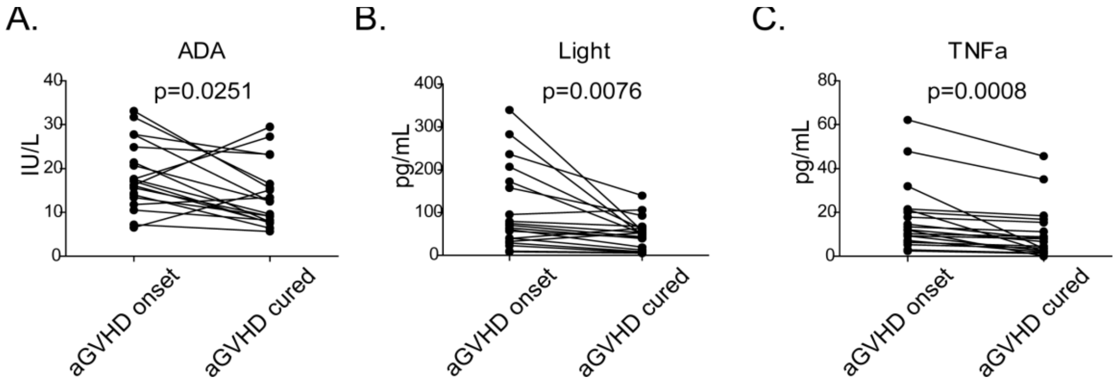

Supplementary Figure 5. The changes in ADA, LIGHT and TNF-α after aGVHD cures.

Supplementary Figure 6.The levels of LIGHT(a), ApoC(b), GGT(c), sCD40L(d), AST(e) and TB in validation group at the day +7 and +14. *P* values were calculated using a Mann-Whitney test. (●, non-GVHD; ■, aGVHD.
